# NKG2A inhibits TH2 cell effector function *in vitro*

**DOI:** 10.1186/1471-2466-7-14

**Published:** 2007-10-10

**Authors:** Robert J Freishtat, Bahar Mojgani, Maryam Nazemzadeh, Kanneboyina Nagaraju, Eric P Hoffman

**Affiliations:** 1 Division of Emergency Medicine, Children's National Medical Center, Washington, DC, USA; 2 Research Center for Genetic Medicine, Children's National Medical Center, Washington, DC, USA; 3 Department of Pediatrics, School of Medicine and Health Sciences, George Washington University, Washington, DC, USA; 4 Georgetown University School of Medicine, Washington, DC, USA; 5 School of Medicine and Health Sciences, George Washington University, Washington, DC, USA

## Abstract

**Background:**

We previously reported that NKG2A, a key inhibitory ligand for HLA-E, is expressed on activated TH2 but not TH1 cells. Here we measured cytokine expression in human *ex vivo *TH2 cells upon activation with anti-CD3/28 and challenge with an NKG2A-specific agonist.

**Methods:**

TH2 cells were purified from healthy volunteers and activated with anti-CD3/28 in the presence and absence of NKG2A-specific agonist. IL-4 was used as a marker of TH2 effector function and measured by flow cytometry.

**Results:**

Activation of TH2 cells increased NKG2A positivity from (Mean ± SE) 7.3 ± 2.4% to 13.7 ± 3.8%; (p = 0.03). The presence of NKG2A agonist did not significantly alter NKG2A expression, however, the percentage of activated TH2 cells expressing intracellular IL-4 decreased from 25.5 ± 6.8% to 9.3 ± 4.8% (p = 0.001).

**Conclusion:**

We show that signalling through NKG2A suppresses TH2 effector function. This may provide a means to modulate Th1/Th2 balance in diseases where Th2 cytokines predominate.

## Background

NKG2A [Swiss-Prot: P26715] is an inhibitory C-type lectin most commonly found on natural killer (NK) cells and cytotoxic T lymphocytes[1-4]. The receptor-ligand binding of NKG2A to MHC class Ib antigen HLA -E[5-10] induces an immunoreceptor tyrosine-based inhibition motif (ITIM) that suppresses effector cell cytotoxic activity[11-14]. It has been reported that this inhibition of cytotoxicity is important in NKG2A's role in the modulation of cytotoxic immune response to viruses[15-20] and cancers[21,22].

Our previous report on NKG2A showed that it is expressed on activated human TH2 but not TH1 cells[23]. Based on our data, we proposed a model where disease states characterized by decreases in HLA-E expression, for example as seen in melanoma[24] and herpes simplex virus infection[25], would lead to decreased agonism at TH2 NKG2A receptors. The resultant decrease in NKG2A inhibitory signaling would lead to relatively increased TH2 cell effector function, exacerbating Th1/Th2 imbalance in diseases where that balance is relevant.

As a first step toward establishing the feasibility of this model, we hypothesized that ligation of NKG2A receptors on activated TH2 cells would be expected to lead to downstream suppression of interleukin (IL)-4 expression[23]. Here, we test this hypothesis using purified human *ex vivo *TH2 cells with activation by anti-CD3/CD28 antibodies and challenge with an NKG2A-specific agonist.

## Methods

### Participants

Apparently healthy, non-atopic, non-asthmatic volunteers between the ages of 18 and 50 years had 60 mL of venous blood drawn directly into ethylenediaminetetraacetic acid (EDTA) following written informed consent. The investigation was approved by the hospital's Institutional Review Board and General Clinical Research Center (GCRC) Advisory Committee and performed in the hospital GCRC.

### TH2 cell isolation

Cell separation procedures were begun within 30 minutes of blood collection. TH2 cells were isolated from whole blood as we previously described[23]. Briefly, EDTA-whole blood was centrifuged at low speed to allow removal of platelet-rich plasma. The remaining cells were diluted and centrifuged over Ficoll Paque PLUS™ (Amersham Biosciences, Piscataway, NJ) density medium to isolate the peripheral blood mononuclear cell (PBMC) layer. The PBMCs were counted by hemocytometer to assure a concentration less than 8 × 10^7 ^cells/mL. TH2 lymphocytes were negatively isolated from the PBMCs using StemSep™ magnetic gravity columns (StemCell Technologies, Vancouver, BC) with a monoclonal antibody cocktail we previously validated for TH2 enrichment to 84% purity[23]. Eluted TH2 cells were immediately placed in cell culture.

### Cell culture

Four cell culture conditions were used (Table 1), including combinations of resting and activated TH2 cells and challenge with an NKG2A agonist. All TH2 cells, except negative controls, were pre-treated with IgG_2a _(R&D Systems, Minneapolis, MN) to prevent non-specific antibody binding. Plates for activated TH2 cell culture conditions were prepared with 10 μg/mL each of plate-bound anti-CD3 (Clone SK7: BD Biosciences, San Diego, CA) and suspended anti-CD28 antibodies (Clone 15E8: Chemicon International/Upstate USA, San Francisco, CA). The enriched TH2 cells (< 1 × 10^6 ^cells/mL) were aliquoted equally into each of the four cell culture conditions suspended in HB 101 Basal Media (Irvine Scientific, Santa Ana, CA), 10% HB Basal Supplement (Irvine Scientific), 10% autologous plasma, 10% Penicillin/Streptomycin (Sigma-Aldrich, St. Louis, MO), and 1% Gentamicin (Sigma-Aldrich). Negative control wells contained TH2 cells in culture medium alone.

**Table 1:** Description of Cell Culture Conditions

**Condition**	**Cell Stimulants (10 μg/mL each)**	Added Antibody* (10 μg/mL each)
Positive Control	anti-CD3 and anti-CD28	None
Anti-NKG2A Antibody	anti-CD3 and anti-CD28	anti-NKG2A
Isotype Control	anti-CD3 and anti-CD28	IgG_2a_
Negative Control	None	None

*Added every 24 hours.

The NKG2A agonist used for these experiments was an anti-NKG2A antibody (Clone 131411, R&D Systems) that specifically binds the NKG2A receptor and elicits its inhibitory signal[26,27]. Cells were treated with either 10 μg/mL of soluble or plate-bound anti-NKG2A antibody as per these previous reports at culture inception and every 24 hours. Results for soluble and plate-bound anti-NKG2A antibody were similar so only results from soluble treatments were used for analysis (Data not shown). Likewise, isotype control wells were treated every 24 hours with 10 μg/mL of anti-IgG_2a _(R&D Systems), the isotype for the anti-NKG2A antibody. All cell culture conditions were maintained for 48 hours at 37°C and 5% CO_2_.

### Flow cytometry

Post-culture TH2 cells underwent a series of incubations with fluorescent-labeled monoclonal antibodies, as well as permeablization and fixation with 2% paraformaldehyde. Four-color flow cytometry (FACSCalibur™ System, BD) was performed with varying combinations of anti-CD3-PerCP (BD), anti-CD4-FITC (BD), anti-CD4-APC (BD), anti-CD8-FITC (BD), anti-NKG2A-APC (R&D Systems), anti-NKG2C-PE (R&D Systems), anti-IFN-γ-FITC (BD), and anti-IL-4-PE (BD) using appropriate isotype and negative (unlabeled cells) controls. Cells were gated for viable TH lymphocytes using forward- and side-scatter properties and CD3+CD4+ double positivity. Flow data were analyzed with FlowJo 7.1 (Tree Star, Inc., Ashland, OR).

### Data analysis

Within- and between-culture condition comparisons were made for percent positive cells and geometric mean fluorescence intensity (MFI). Statistical significance was tested with SPSS 13 (SPSS, Chicago, IL) using paired T-tests.

## Results

A total of ten apparently healthy, non-atopic, non-asthmatic adult volunteers were enrolled in this study. Both genders and a diverse group of ethnicities and races were represented among the participants.

Following culture in the four distinct conditions (positive control, anti-NKG2A antibody, isotype control, and negative control) TH2 cell samples were examined by four-color flow cytometry to confirm the expected rise in cells positive for NKG2A surface expression. The cultured cells were > 97% pure CD3+CD4+CD8- TH cells. We found significant up-regulation of TH2 NKG2A positivity from 7.3 ± 2.3% in negative controls to 13.7 ± 3.8% in positive controls (p = 0.03) consistent with our previously published data[23]. There was no significant difference in the surface expression of NKG2A (16.0 ± 4.0%) on the activated TH2 cells cultured in the presence of anti-NKG2A antibodies and the positive controls. Results for soluble and plate-bound anti-NKG2A antibody were similar so only results from soluble treatments were used for analysis. Additionally, there was no appreciable change in the low level expression of activating NKG2C (data not shown). This was also consistent with our previous findings[23].

**Figure 1:**
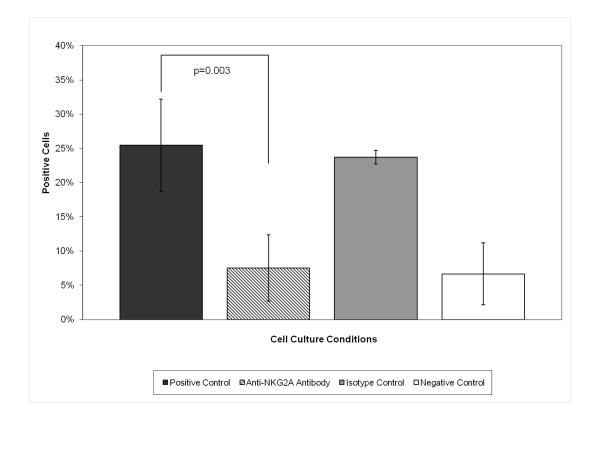
**Comparison of intracellular IL-4 expression between culture conditions**. Comparison of TH2 lymphocytes cultured in each of four culture conditions. Mean ± SEM percent cells positive for intracellular IL-4 is shown in this graph.

**Figure 2:**
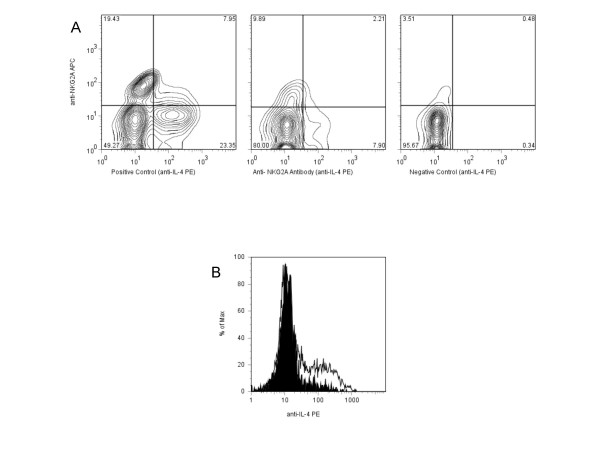
**Representative flow cytometry comparison of surface NKG2A and intracellular IL-4 co-expression between culture conditions**. Flow cytometry comparison of NKG2A and IL-4 co-expression for an exemplary sample of TH2 cells cultured with CD3/CD28 antibodies (positive control) and soluble CD3/CD28/NKG2A antibodies (A). The accompanying histogram shows the similar decrease in overall IL-4 expression in the same positive control (outlined) and Anti-NKG2A samples (shaded) (B). Analyses were gated by forward and side-scatter properties and on CD3+CD4+ cells.

In order to assess the impact of NKG2A signaling on TH2 cell effector function following activation through the TCR, we performed flow cytometry for intracellular expression of IL-4 and IFN-γ. There was significant up-regulation of intracellular IL-4 from 6.7 ± 4.6% in negative controls to 25.5 ± 6.8% in positive controls (p = 0.003). (Table 2 and Figure 1) When TH2 cells were activated in culture in the presence of anti-NKG2A antibodies, the intracellular expression of IL-4 fell from 25.5 ± 6.8% to 9.3 ± 4.8% (p = 0.001) overall. (Table 2 and Figure 1) This level of IL-4 expression was not significantly different from the negative controls. As before, results for soluble and plate-bound anti-NKG2A antibody were similar so only results from soluble treatments were used for analysis. Additionally, double-staining of activated TH2 cells for surface NKG2A and intracellular IL-4 showed the reduction in IL-4 expression occurs mainly among the NKG2A positive cells. (Figure 2)

**Table 2:** Between Cell Culture Condition Comparisons of TH2 Cell Positivity for IL-4 and IFN-γ

**Condition**		**IL-4 Positive (%)**	**Fold IL-4 MFI**	**IFNγ Positive (%)**	**Fold IFNγ MFI**
Positive Control (CD3/CD28)	Mean	25.5%	22.47	7.7%	7.43
	SEM	6.8%	12.26	1.3%	1.42
	*p-value (Negative Control)*	**0.003**	**0.03**	**0.05**	**0.05**
	*p-value (Isotype Control)*	0.11	0.18	0.35	0.24

Anti-NKG2A Antibody (CD3/CD28 + anti-NKG2A)	Mean	9.3%	4.39	5.5%	5.29
	SEM	4.8%	1.81	1.3%	1.50
	*p-value (Positive Control)*	**0.001**	**0.01**	**0.04**	**0.01**
	*p-value (Isotype Control)*	**0.005**	0.066	0.427	0.234

Isotype Control (CD3/CD28 + IgG2a)	Mean	23.7%	7.81	4.9%	6.50
	SEM	1.0%	0.72	2.7%	0.43
	*p-value*	-	-	-	-

Negative Control (Culture Medium Alone)	Mean	6.7%	2.76	4.8%	4.83
	SEM	4.6%	0.63	0.8%	1.60
	*p-value*	-	-	-	-

p ≤ 0.05 in **bold**.

We also examined expression of IFN-γ and found similar changes in intracellular expression. Negative control expression was 4.8 ± 0.8%. This increased to 7.7 ± 1.3% in positive controls (p = 0.05) and fell to 5.5 ± 1.3% in the presence of anti-NKG2A antibody (p = 0.04). This level of expression was not different from negative controls.

## Discussion

Using a monoclonal antibody cocktail we developed for the negative isolation of human TH2 cells, we confirmed our previous work showing a significant increase in NKG2A surface protein expression following the activation of TH2 cells[23]. Here, we tested the hypothesis that activated TH2 cell effector function is diminished in the presence of NKG2A signaling.

We induced NKG2A signaling using a monoclonal antibody specific for the NKG2A receptor that has been shown to be an agonist and cause suppression of NK cell cytotoxicity in P815 target cells[27]. We used a magnetic gravity column isolation to obtain quiescent human peripheral blood TH2 cells. These TH2 cells were bound with the NKG2A agonist antibody and then studied for downstream signaling of CD3/CD28. Activated NKG2A+ TH2 cells cultured with this NKG2A agonist showed a significant decrease in effector function indicated by intracellular IL-4 expression measured by flow cytometry. As NKG2A is expressed on TH2 but not TH1 cells [23], our ligation of NKG2A would have the functional consequence of altering Th1/Th2 cytokine ratios. To our knowledge, this is the first *ex vivo *immune modulation of human TH2 cells.

The data presented here suggest that use of this NKG2A agonist antibody could modulate Th1/Th2 balance toward Th1 and thus modulate inflammatory responses. Consistent with this model, Kawamura, et al. used anti-NKG2A monoclonal antibodies to restrict donor T cell expansion and suppress inflammation in a murine model of acute graft-versus-host disease[28]. Our data suggest that this may also be possible in human diseases like asthma, colitis, and autoimmune diseases where Th1/Th2 cytokine balance is important.

Our finding that the NKG2A receptor is capable of modulating TH2 cell effector function is analogous to its purpose on CD8+ cytotoxic T cells, where it helps prevent cytotoxicity of HLA-E expressing cells. In TH2 cells it appears that there is concomitant binding of NKG2A to HLA-E along with the CD4+-TCR to MHC class II. This parallel receptor-ligand binding likely results in transmission of an inhibitory signal from NKG2A via SHP-1 and SHP-2 along with the activating signal from the TCR. Given the lack of NKG2A on activated TH1 cells, this could lead to a relatively robust TH2 response.

As in our previous study, we continued to find sizeable inter-individual variability in the level of NGK2A expression before and after TH2 cell activation. This has been described previously for NKG2 receptors in rhesus monkeys [29] and is either indicative of dynamic switching of NKG2 receptor expression between subtypes or individual genetic differences.

## Conclusion

In conclusion, we identified significant suppression of IL-4 expression in activated TH2 cells via NKG2A binding and ultimate TCR signaling inhibition. Extrapolated to clinical scenarios where HLA-E and thus NKG2A binding are in low abundance, we have shown that TH2 cells could be expected to exhibit a relatively robust response and that this response could potentially be modulated by NKG2A agonist. This represents a new aspect of Th1/Th2 balance in inflammation with possible clinical implications.

## Abbreviations

TH – T-Helper Cell; IL – Interleukin; IFN – Interferon; TCR – T Cell Receptor; PBMC – Peripheral Blood Mononuclear Cell

## Competing interests

The author(s) declare that they have no competing interests.

## Authors' contributions

RJF conceived of the study and participated in its design and coordination. KN and EPH participated in the design of the study. RJF, BM, and MN carried out the cell culture and flow cytometry studies. RJF performed the statistical analyses. RJF, KN, and EPH drafted the manuscript. All authors read and approved the final manuscript.

## Pre-publication history

The pre-publication history for this paper can be accessed here:


